# An Effective YOLOv11 Grain Detection Model Trained on Intact Barley Spikes Reveals a QTL Containing a Pivotal Regulator of Lateral Spikelet Formation

**DOI:** 10.3390/plants15101518

**Published:** 2026-05-15

**Authors:** Brittany Clare Thornbury, Chengdao Li

**Affiliations:** 1 Western Crop Genetics Alliance, College of Science, Health, Engineering and Education, Murdoch University, 90 South Street, Murdoch, WA 6150, Australia; bcrobertson97@gmail.com; 2 Western Australian State Agricultural Biotechnology Centre, Murdoch University, 90 South Street, Murdoch, WA 6150, Australia; 3 Department of Primary Industries and Regional Development, 3-Baron-Hay Court, South Perth, WA 6151, Australia

**Keywords:** grain number, yield, barley, phenotyping, machine learning, detection, YOLO

## Abstract

Grain number is a primary agronomic trait for targeted yield improvement, with the prospect of enhanced grain production leading to greater food security. Given the complex polygenic nature of the grain number trait, large sample sizes are essential for effective QTL identification. The implementation of trained computer vision models for grain detection offers a timely and cost-effective solution for rapid QTL isolation. In this study, we trained a grain detection model using Ultralytics’ You Only Look Once (YOLOv11) framework. Training was completed on 1000 images of barley spikes, derived from a doubled haploid (DH) population descended from Hindmarsh and RGT Planet. The trained model, termed BarleyGC, achieved satisfactory accuracy metrics (mAP50–95 = 71.9%, recall = 96.7%, precision = 97.1%). Phenotypic characterisation of the DH population was completed with BarleyGC on a distinct collection of 973 images. The Pearson correlation coefficient (r) between model and manual-derived counts for the trait of grain number per spike was 0.895 (*p* < 0.0001), and 92.4% of all measurements fell within three grains of the manual measurement. Downstream QTL analysis on the phenotype data (*n* = 153 DH lines), revealed a QTL peak at position 224.959 cM on the genetic map (LOD = 3.14), named qGN-2H. The QTL region contained 21 candidate genes—including HORVU2Hr1G092290 (HORVU.MOREX.r3.2HG0184740), encoding the six-rowed spike 1 (*Vrs1*) gene—a well-characterised major regulator of row-type divergence and lateral spikelet development. Our study demonstrates the power of the YOLOv11 framework for grain quantification, with BarleyGC capable of grain detection directly from images of intact spikes in two-rowed barley varieties—thus achieving accelerated sample processing for the grain number trait.

## 1. Introduction

Improving the capacity of barley (*H. vulgare*) varieties for enhanced grain output is a major endeavour, given its position as the fourth most widely cultivated cereal crop globally [1]. Further underpinning the target for improved yield, is the likelihood that future farming practices will shift to increase outputs over smaller land areas. This is in part due to a combination of climate induced field impacts and an increased pressure to restore agricultural lands for biodiversity purposes [2,3]. Improvement of crop production can be attained through a variety of avenues, including appropriate water supply, soil management, and fertiliser-based supplementation. However, each strategy comes with their own set of challenges. Such obstacles include a future shaped by limited water availability, due to increasingly frequent reductions in seasonal rainfall. This is especially evident in susceptible climate regions such as the agricultural lands of Western Australia, predicted to experience barley yield losses >30% in northern growing regions by 2050 [4]. The urgency to improve yield potential is further exacerbated by Australia’s dominance of the global barley trade, accounting for up to 40% of global malt quality barley exports [5].

Genetic improvement of barley lines and other cereals has become a major focus for surpassing yield targets in recent years. The central advantage is that high yield potential is retained in the genetic signature for subsequent generations, thus reducing reliance on field modifications. Barley yield is underpinned by the two key traits of grain number and grain weight. A relevant study by Serrago et al. (2025) investigated both major yield traits and their fluctuation as influenced by underlying genetic and environmental influences, using a compilation of research data spanning over 25 years (1996–2021) [6]. Authors identified grain number trait as the major determinant of grain yield, explaining 86% of the variability in grain yield when compared with grain weight, which accounted for only 13% of yield variability [6]. In turn, this highlights the major relevance of grain number as a primary indictor of yield capacity in barley.

Machine learning is a useful tool for accelerated phenotyping of agronomic traits [7]. The implementation of trained phenotyping pipelines greatly reduces the time required for sample processing, in addition to limiting the associated costs involved with human labour required for phenotyping tasks [7]. Numerous sophisticated models have been reported in the literature and are used across a range of agricultural species for purposes ranging from disease detection and susceptibility, crop environmental resistance, soil quality, and multi-trait phenotyping from sample images [8,9,10]. Convolutional neural networks (CNNs), demonstrate high plasticity and rapid adaptability across diverse image collections, making such constructs appealing for a range of phenotyping tasks [11]. The You Only Look Once (YOLO) object detection framework is one example that is widely utilised in the agronomic space, favoured for both processing speed and accuracy [11,12]. Examples of YOLO framework deployment include the use of YOLOv8 in the detection of peanut yield losses, and Yolov5 to detect early disease in rice and differentiate weeds from crops in aerial images [13,14,15].

Several phenotyping protocols adopting computer vision models, such as track-based systems, rely on sophisticated imaging setups—in many cases, requiring complex assembly in the field [16,17]. Such technology, in particular 3-dimensional image capture platforms, may cost up to 30,000 USD which poses accessibility concerns [16,18]. The principal aim of this study was to develop a simple, rapid and low-cost grain number quantification workflow in two-rowed barley using YOLOv11. Model utility to effectively detect grains was measured following deployment on phenotype data consisting of 973 barley spike images. The derived grain number per spike data was used to identify quantitative trait loci (QTLs) significantly associated with the grain number trait. Barley spike images used for training and phenotyping, were captured from a doubled haploid (DH) population developed from the Australian feed variety Hindmarsh and elite European malting variety RGT Planet. Any significant genetic loci found within identified QTL region(s), may provide novel avenues for expanded yield capacity in barley.

## 2. Results

### 2.1. YOLOv11 Based BarleyGC Allowed for Accelerated Phenotyping of Barley Grain Head Samples

**Figure 1:**
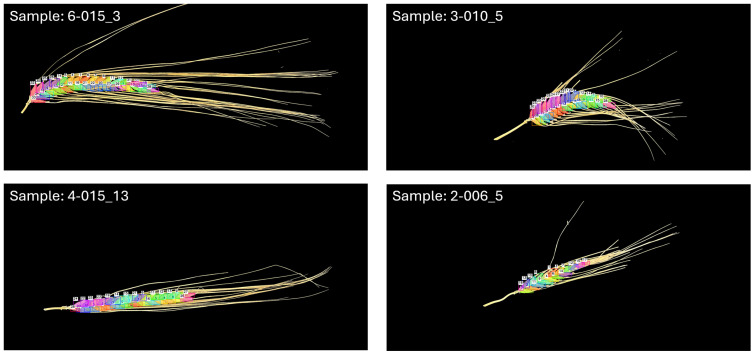
Debug visualisation of trained detection model performance on four grain head sample images (labelled in the top left) derived from the DH population. Bounding boxes of grains identified by the trained ‘BarleyGC’ are colourised and labelled in order of detection. Segmentation masks of the grains (generated with SAM2) are highlighted in the same colour as the bounding boxes of unique grain detections. The first part of the sample name (before the underscore, ‘_’) denotes the unique plot number to which the barley head sample is derived, and the number following the underscore is the number order in the image collection from the sample plot.

In this study, we developed a grain detection model for the accelerated phenotyping of barley head samples collected from our DH barley population. Following the final training run, the model achieved a precision of 97.1%, recall of 96.7%, and mAP50–95 of 71.9%. These metrics indicated that the model was effective at grain detection, achieving accuracy above 70% even when intersection over union (IoU) thresholds become exceedingly stringent (requiring predicted bounding box and ground truth overlap of up to 95%). The mean average precision was an additional indicator of the model’s general effectiveness at grain object detection, performing at 98.9% accuracy when the IoU threshold only required a 50% overlap between ground truth and predicted bounding boxes. For phenotyping purposes, BarleyGC was implemented in tandem with SAM2 to generate bounding box annotations and segmentation masks of grains for 973 barley head images. Figure 1 shows the debug output of four barley head images phenotyped with our auto annotation pipeline. The debug output illustrates the effectiveness of BarleyGC, demonstrating elegant grain detection capabilities across a range of phenotypes—including straight and curved grain head architectures, represented by images 4-015_13 and 3-010_5 respectively (Figure 1). Annotation pipeline effectiveness was reduced for barley head samples with awns overlapping grains—for example, in image 6-015_3 (Figure 1). However, the number of trained BarleyGC detections produced for image 6-015_3 was the same as the manual grain number (33), with SAM2 annotations producing an error size of four grains. This was an artefact of segmentation masks splitting into separate grain annotations at the border of the awn. An additional caveat of our trained detection model was its difficulties in identifying grains in barley heads comprising overlapping/reduced visibility grains, particularly near the tip of the head; at times, it failed to detect grains with clear representation (Figure 1, image 2-006_5).

**Figure 2:**
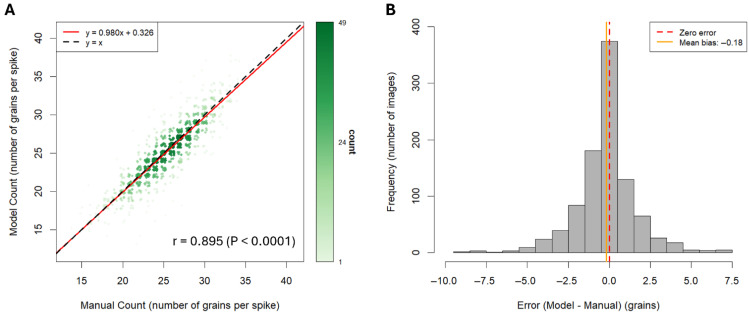
Scatterplot (**A**) with fitted linear regression line (red), showing the correlation between model and manual counts for grain number (number of grains per spike), for a collection of 973 images of barley heads. Included in the scatterplot is a black dashed regression line (y = x), representing a hypothetical perfect agreement in measurements, for comparison to the fitted red regression line, and the Pearson’s correlation coefficient (r) (and its *p*-value). A density gradient is presented to the right, with increased opacity regions corresponding to grain numbers with higher data point frequencies, as calculated via 2D kernel density estimation. Plot (**B**) shows a frequency histogram of error values (model—manual grain numbers) for the image collection. Lines representing an error value of 0 (red dashed line) and the mean error bias (orange) are plotted above.

An investigation of the model’s phenotyping accuracy was additionally completed, to determine how closely BarleyGC performed relative to the manual grain numbers. We calculated the Pearson correlation coefficient (r), to measure the extent of correlation between the manual counts and model predictions for grain number. An r value of 0.8948 (*p* < 0.0001) indicated a high degree of correlation between the model and manual counts across the image collection used for phenotyping (*n* = 973) (Figure 2A). The coefficient of determination (R^2^) was 0.8007, indicating that the model accounted for ≈80% of the variability observed in terms of the relationship between the manual and model-generated grain numbers. Linear regression analysis was performed on the manual and model-predicted grain number data, revealing a slope of 0.9798 and intercept of 0.3265 (Figure 2A). In turn, this indicated that the model was highly concordant with manual counts and scaled appropriately with variations in phenotype data, adding an average of 0.98 grains for each additional grain accounted for in the typical phenotyping process. A mean error bias of −0.18 was indicative that model behaviour tended to undercount grains, albeit by an exceedingly small margin. The mean absolute error (MAE) showed that the model deviated from manual counts by an average of 1.2 grains, further reflecting effective model phenotyping performance. Error size ranged from −9 grains to 7 grains (standard deviation = 1.86 grains). The majority of model predictions (70.4%) fell within one grain, 85.7% fell within two grains, and 92.4% of model predictions fell within three grains of the manual measurement (Figure 2B). Overall, the model demonstrated excellent grain detection across a range of barley grain head presentations. Despite some instances of reduced model efficiency at generating grain annotations (especially for images with overlapping awns and partially obscured head presentations), the error size remained minimal.

### 2.2. A High Degree of Spatial Heterogeneity Observed Across Plots for the Grain Number Trait

**Figure 3:**
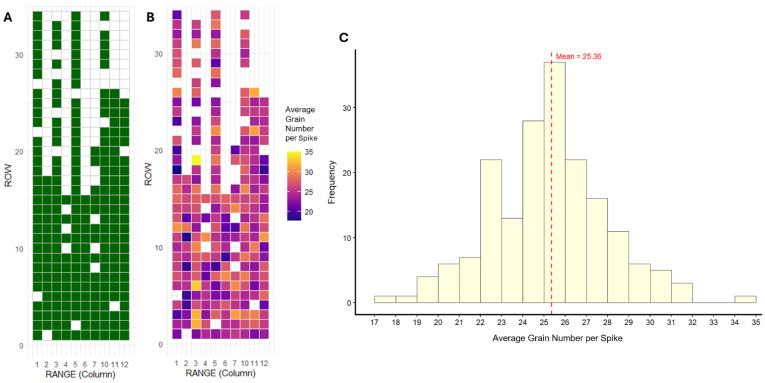
Field occupancy map (**A**) showing all 229 sampled DH plots included in the initial assessment of phenotype data for spatial correction, as organised in the original field layout at the Wongan Hills trial site. The plot to the right (**B**) shows the same field occupancy arrangement as a heat map for grain number, enabling visualisation of any potential spatially relevant phenotype clusters. Plot (**C**) illustrates a frequency histogram of median-adjusted phenotype data, showing line counts on the *y*-axis for average grain number measurements (*x*-axis) collected for 183 DH barley lines in the study population. The mean grain number is plotted as a dotted red line.

Prior to QTL identification, we investigated the phenotype data collected for the DH population, to determine any spatially influenced phenotype signals that may have later confounded downstream analysis. Our initial phenotype data set comprised 229 sampled plots, where the plot distribution is shown in Figure 3A. The heat map of the average grain number per spike across plots indicated that phenotypic measurements were spatially heterogeneous, with no clear clustering of adjacently similar phenotypes within the field (Figure 3B). The 229 sampled plots in this study comprised 183 unique genotypes, with a replication rate of 22.4% (41 lines being replicated). Of the repeated genotypes, 36 were duplicated and five were plotted in triplicate. The mean coefficient of variation (CV) was determined to be 0.084 and 0.107 with average ranges of 3.01 and 5.06 grains for two- and three-plot replicates, respectively, indicating relatively low average phenotype variability (Table 1A). Next, the repeatability value was calculated to determine the correlation between duplicate plots, using Pearson’s correlation coefficient. Repeatability was negative (−0.222), indicating that some genotype pairs were displaying somewhat opposing values at duplicate plots (Table 1B). Six outliers were identified among duplicate plots, with phenotype differences ranging from 6 to 12.6 grains per spike. Once outliers were removed, the repeatability value improved to 0.397—however, this value still indicated that a substantial portion of the variation between phenotypes (approximately 60%) was due to other random effects (Table 1B).

An assessment of calculated variance components revealed that the majority of observed field variation (84.7%) was due to residual (random) effects, with a smaller spatial contribution of 15.2% and substantially low genetic contribution of 0.1%. Furthermore, the plot-level broad-sense heritability (H^2^) was minor, at 0.12%, indicating a minimal genetic signal driving observed phenotypic variation in plot values. It was also determined that within-genotype standard deviation (between replicate plots) and between-genotype standard deviation (between genetically distinct plots) were similar, at 2.96 and 2.76 respectively, thus complicating isolation of a genetic signal from plot-wise environmental effects.

Given that the field-derived phenotype data exhibited substantial stochastic noise with limited evidence of major spatial influence on observed values, the final dataset was prepared such that simple means were used for majority of replicated genotypes. For replicate plots that differed by more than five grains (*n* = 8), the median was instead used for reduced impact of residual phenotype variability on QTL analysis. Figure 3C shows the histogram of median-adjusted phenotype data (*n* = 183 genotypes). Grain number measurements of barley head samples followed a normal distribution (*p* = 0.546; W = 0.993 following the Shapiro–Wilk test), ranging from 17.8 to 35 grains, indicating suitability for downstream QTL analysis, with a mean grain number of 25.36 and standard deviation of 2.77.

### 2.3. Population Structure of Doubled Haploid Progeny Revealed Three Distinct Genetic Clusters That Minimised the Initial QTL Signal

**Figure 4:**
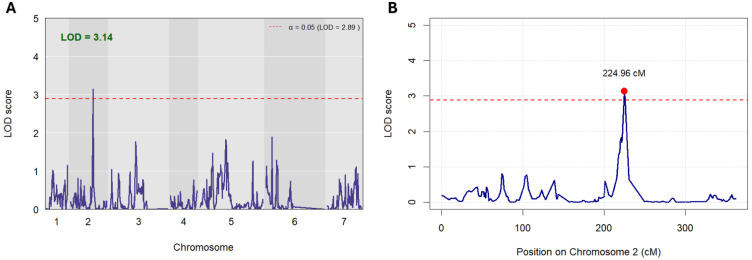
Plots illustrating scan results following QTL analysis on the DH population, consisting of 153 unique DH lines. PC corrected QTL peaks are plotted (**A**), using the first 5PCs as covariates. The Logarithm of the Odds (LOD) threshold for significant QTL(s) (LOD = 2.89) is plotted as a dashed red line, and was determined using permutation testing with 1000 permutations, at a significance of α = 0.05. The maximum LOD score identified following analysis is plotted in green above. Plot (**B**) illustrates a zoomed view of the statistically significant marker located on chromosome 2, at position 224.96 centimorgans (cM). The LOD score threshold for statistical significance is plotted above (dashed red line).

**Figure 5:**
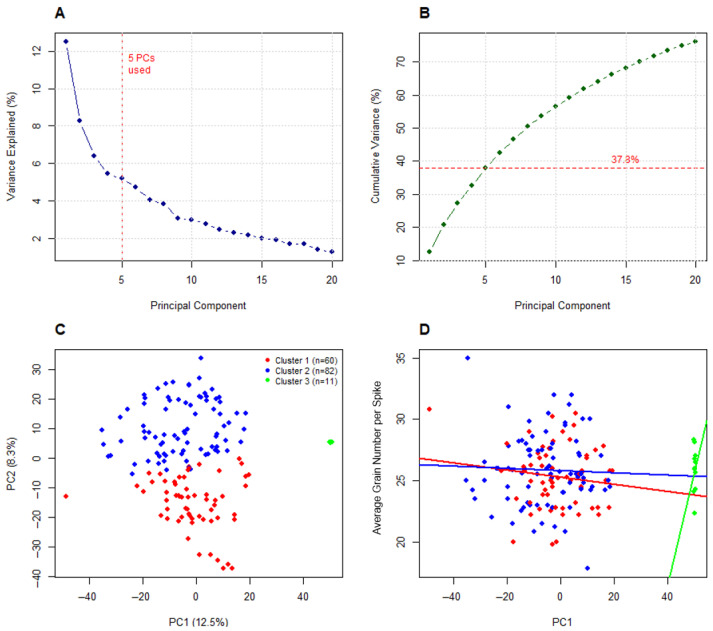
Panel figure illustrating various elements of principle component correction for population structure in the doubled haploid (DH) barley population, derived from a parental cross of Hindmarsh and RGT Planet. (**A**,**B**) show a scree plot and cumulative variance plot, respectively. (**C**) shows the clustering pattern of underlying population structure in the DH population, as plotted by the first two principal components, with sub-populations colourised in the legend. (**D**) shows the first principal component (*X*-axis) as plotted against average grain number per spike measurements for the 153 barley lines. Included in plot (**D**) are regression lines for the three population clusters.

Following the initial scan, a single QTL for grain number was identified on chromosome 2H. The QTL, named qGN-2H, possessed an LOD score of 1.82, which was below the permutation-derived significance threshold (LOD = 3.02 or greater) (Supplementary File, Figure S1A). In turn, the DH population was investigated to determine any sources of population structure that may be reducing the QTL peak. Kinship analysis was conducted, revealing an average pairwise kinship of 0.512 in the DH population. A revised QTL scan was subsequently completed, this time incorporating associations from kinship matrices generated by the LOCO method, via a linear mixed model (LMM) approach. Following kinship-based correction, the LOD score was 2.03 (below the kinship-adjusted LOD of 2.83 derived from permutation testing) (Supplementary File, Figure S1C). Finally, principal component analysis (PCA) was performed, using the first five PCs as covariates in the revised QTL scan. Following PC-based correction, the original QTL peak detected during the initial scan improved above the significance threshold (LOD = 2.89), with an LOD score of 3.14 (Figure 4A). The QTL qGN-2H was located at 224.959 cM based on genetic map positioning (Figure 4B). The choice of five principal components was considered suitable given they captured substantial variance (37.8%) with diminishing individual contributions (less than 5%) for components beyond 5PCs (as evidenced by the scree plot in Figure 5A). Although 3PCs effectively captured the structural nature of the DH population during clustering analysis (Figure 5C), PCs 4 and 5 were also incorporated for QTL detection, to capture any residual structural variation that may have been omitted.

Figure 5 above shows the results of PCA and clustering analysis (using K-means), where the first five PCs captured 37.8% of the total variance in the genotype (marker) data (Figure 5A,B). The First two principal components were additionally plotted and colourized by k-mean-assigned clusters (Figure 5C). Three distinct genetic subclusters were present in the DH population—these being cluster 1 (*n* = 60), cluster 2 (*n* = 82) and cluster 3 (*n* = 11) (Figure 5C). As noted above, the presence of distinct population clusters further supported the use of PCA correction for an improved QTL detection signal. A visual assessment of regression lines illustrating the relationship between grain number and PC1, showed variable impacts of population structure on phenotype in different clusters, as evidenced by the near flat regression line of cluster 2, relative to the moderate-to-weak negative and strong positive slopes of clusters 1 and 3, respectively (Figure 5D).

The QTL qGN-2H was defined by colocalizing markers m574 and m575 in the physical map, exhibiting 100% genotype concordance across the DH population. The marker m575 was selected for downstream QTL analysis of qGN-2H, due to possessing more complete genotype data (allele data was present for four extra markers versus m574). ANOVA was subsequently performed, to determine whether the QTL effect of qGN-2H remained relevant within clusters and was thus not confounded by genetic background influences. Two linear models were compared, with model 1 predicting grain number variation due to genetic cluster assignment, and model 2 additionally incorporating qGN-2H in the prediction of grain number phenotype. The results of the ANOVA showed that qGN-2H significantly improved model capacity to predict grain number across all three clusters (*p* < 0.001) (Table 2). Hence, it was determined that the effect of qGN-2H was not a byproduct of clustering behaviour within the study population.

### 2.4. The RGT Planet-Derived ‘A’ Allele Imparts a Favourable Effect on Grain Number in DH Progeny at qGN-2H

**Figure 6:**
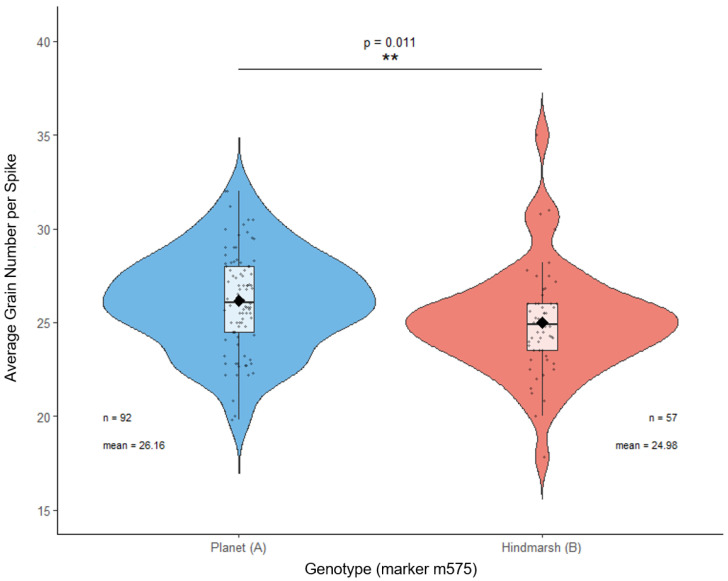
Plotted distributions of grain number measurements for the RGT planet-derived A allele and Hindmarsh parent-derived B allele, at significant marker position m575 defining qGN-2H. A total of 149 DH lines used in QTL identification had available genotype data for allelic segregation analysis. Violin plots include box and whisker plots with median (line) and mean (black diamond) additionally included. Sample size for both allele classes, and their respective means, are plotted above. A *p*-value of 0.011, describing the difference in distributions of grain number for the parent alleles, was considered to be highly significant (**) in this study.

Allelic segregation at qGN-2H (marker m575) was investigated in the DH population, with 92 DH lines possessing the RGT planet-derived A allele and 57 lines possessing the Hindmarsh-derived B allele at the marker position. The difference in grain number distributions for the respective homozygous genotypes at qGN-2H was statistically significant (*p* = 0.011). The A allele distribution exhibited an average grain number of 26.16, and the B allele distribution possessed an average grain number of 24.98, giving an effect size of 1.18 grains for those possessing the A allele (Figure 6). Finally, the phenotypic variance explained was determined to be 7.9%, thus classifying qGN-2H as a moderate effect QTL for grain number in this study.

### 2.5. The qGN-2H QTL Region Contains the Candidate Gene Vrs1, a Major Regulator of Grain Number in H. vulgare via Transcriptional Modulation of Lateral Spikelet Formation

Using the Morex V1 assembly (Genome build: GCA_901482405.1), the qGN-2H QTL region was searched to identify potential candidate genes supporting the observed QTL impact on grain number in the DH population. A total of 21 candidate genes were identified and found to exhibit a diverse array of biological roles following CD-Search-based prediction of protein function. Of the candidates present in the QTL region, six genes presented ambiguous, undefined or absent domain architecture (Table 3). Of those 15 genes with predicted domain architectures, candidates were inferred to participate in a range of processes—including, but not limited to—transcriptional regulation, respiration, stress response, and growth (Table 3). The candidate genes HORVU2Hr1G092290, located at 652.03 Mb on chromosome 2H, and HORVU2Hr1G092460, located at position 653.53 Mb on chromosome 2H were of principal interest for further functional investigation. This was due to their roles as transcriptional regulators. In addition, the QTL marker m575 was located within close proximity of HORVU2Hr1G092460, with the gene start site located within ~1 Kb of the marker. In fact, the HORVU2Hr1G092460 gene region (653,533,189 bp–653,535,350 bp) included the m575 marker, which was located at 653,534,354 bp on the physical map. HORVU2Hr1G092460 encoded a protein product containing a B-box zinc finger domain, which is noted to participate in photo-regulatory modulation of flowering development (Table 3, see references). Given the intrinsic relationship between flowering regulation and grain development, HORVU2Hr1G092460 was considered a prime candidate to support observed improvements to grain number phenotype imparted by qGN-2H. In turn, the transcriptional profile of HORVU2Hr1G092460 across the barley pangenome was interrogated using the PanBARLEX pangenome database, to identify topological sources of candidate gene expression for the equivalent pangenome sequence cluster (BarleyCDS90_22767). The pangenome tissue-expression profile for HORVU2Hr1G092460 was shown to be the highest in the root (with total expression being 27.52 transcripts per million (TPM) across all genes, with low to background levels of expression observed for the Shoot (5.11 TPM) Inflorescence (1.65 TPM) and Caryopsis (0.97 TPM) (Supplementary Figure S2B). This pattern of expression suggested that candidate gene HORVU2Hr1G092460 likely had an alternative role in transcriptional regulation. HORVU2Hr1G092460 was thus no longer considered a primary candidate for grain number regulation in this study, given its low expression in floral organs and photo-exposed plant regions.

Given its homeobox domain, HORVU2Hr1G092290 was additionally considered a candidate of interest for grain number in this study. A UniProt BLAST search using the HORVU2Hr1G092290 amino acid sequence, revealed a top hit with entry B8PYL4. The entry described a Homeobox-leucine zipper protein *Vrs1* (six-rowed spike 1), a well-characterised regulator of lateral spikelet fertility, sharing 100% identity with the query sequence. Based on its latest MorexV3 annotation (HORVU.MOREX.r3.2HG0184740), the candidate gene was submitted to the PanBARLEX database (sequence cluster BarleyCDS90_05674) to further assess its pangenome expression profile. As expected for a major regulator of row-type/grain formation, tissue-specific expression was the greatest in the Inflorescence, with mean pangenome expression of 16.03 TPM (Supplementary Figure S2A). Of particular interest, was the low-level expression of *Vrs1* in RGT planet relative to the majority of pangenome varieties, with a mean expression level of 3.31 TPM. Overall, the qGN-2H QTL region revealed an expansive collection of gene candidates for grain number regulation, with inferred diverse functionality. HORVU2Hr1G092290, encoding the extensively characterised *Vrs1* gene, was selected as the primary candidate supporting the observed impact of qGN-2H on grain number observed for DH progeny.

## 3. Discussion

### 3.1. Population Structure Interference Is a Likely Result of Upstream DH Generation Protocol—Leading to Selective Retention of Genotype Clusters Based on Survivability

In this study, the strength of grain number QTL qGN-2H was initially below the statistical threshold for significance (LOD = 1.82). Following revised QTL analysis with kinship correction, there was a marginal improvement to the peak LOD score (2.03). However, a substantial improvement to the LOD score was observed following PC-based correction for population structure (LOD = 3.14). Principal component analysis subsequently revealed three distinct population clusters in our DH population. These elements of population structure indicated that selective pressures, most likely present during the microspore phase of anther culture protocol, led to the retainment of favourable genotype combinations related to survivability. We speculate that this phenomenon likely substantiates the observed capacity of PC-based QTL correction at conserving the QTL signal, given the PCs capture variation across discrete population substructures. This may explain the lower signal preservation following kinship-based QTL correction, which conservatively models confounding population structure as continuous in nature. As a result, kinship correction may potentially absorb meaningful QTL signal [34]. In spite of this, the LOD peak position remained the same across all three QTL identification methods, highlighting the robustness of the qGN-2H locus (Supplementary File, Table S1).

Anther culture has emerged as a rapid and effective tool for in vitro DH population development in *H. vulgare*. The process involves the forced transformation of microspores (haploid reproductive units) from a gametogenic state towards embryogenesis. This leads to the eventual formation of diploid plantlets carrying a full homozygous complement of the microspore progenitor [35]. Diploid attainment may occur via spontaneous generation, or via chemical mediation methods (i.e., colchicine treatment) [36]. Embryogenesis induction uses several stress-inducing methods, including heat and cold shock, or nutrient limitation. Genetic selection of plantlets exhibiting resistance to these introduced stressors, is commonly an innate byproduct of the anther culture process [35,37]. Indeed, it is widely established that specific barley genotypes are genetically more amenable to anther culture methods, due to the possession of polymorphisms enabling enhanced survivability under the various treatment steps. For example, one study showed greater capacity for two-rowed barley cultivars to generate larger DH populations, versus six-rowed genotypes [38]. In addition, winter type barley has been shown to demonstrate superior survivability to microspore culture treatments, in part due to the possession of polymorphisms for improved stress response [39]. Given that DH progeny are generated from a heterogeneous F1 population of hybrids originating from a cross of two genetically distinct parents (in our case, Hindmarsh and RGT Planet), the genetic sources enabling survivability are particularly limited. In one such study, authors noted higher segregation distortion in a DH population derived from an F_1_ generation resulting from a cross of Tadmor and WI2291, relative to the non-DH F_2_ generation. The DH population showed prominent segregation distortion on Chr4, favouring polymorphisms from the WI2291 variety, which demonstrated superior survivability during the plantlet regeneration step of microspore culture protocol [40]. Thus, the observed high proportion of average shared alleles between DH pairs in our study, reflected the likely genotype profile enabling survival during DH development of our QTL mapping population. Finally, we predict that this genotype profile further diverged into three distinct sub populations (clusters) in our DH line collection, each reflecting a unique genetic survival strategy (Figure 5).

### 3.2. BarleyGC Offers Robust Grain Detection on Phenotypic Datasets with Reduced Sample Processing Requirements

Machine learning techniques were used during the phenotyping phase of our study, leading to the development of a simple grain detection model using the YOLOv11 framework, termed ‘BarleyGC’. BarleyGC enabled accelerated the processing of over 970 unique barley head samples, exhibiting a range of phenotypes (Figure 1). As reported in Section 2.1, BarleyGC demonstrated excellent accuracy metrics, with high levels of correlation for manual and model counts (r = 0.8948; *p* < 0.0001), and over 90% of model-predicted counts falling within 3 grains of the observed phenotype, across all samples. These features are somewhat comparable to GrainNet—a recently developed and highly effective model for grain detection in wheat, developed by Wang et al. (2025) and built using feature extraction derived from the YOLOv7 framework [41]. GrainNet was developed on a training dataset of wheat grain samples of diverse genotypic origins, adhesion levels, and backgrounds, leading to a final R^2^ value of 0.93 and MAE of 5.97, versus an R^2^ of 0.80 and MAE of 1.86 observed for our barley grain detection model [41]. Thus, BarleyGC exhibited promising performance, approaching that of state of the art, modern grain detection models.

One desirable feature of BarleyGC is its development using barley head samples taken directly from the field with no further processing, thus proving increasingly practical for real-world deployment. Numerous high-performing grain detection models are trained on phenotypic image sets including pre-threshed grains—that is, those already separated from the seed head [41,42,43,44]. In contrast, our study has developed an effective barley grain detection model capable of efficient grain number quantification from the barley head directly. This in turn offers a substantial reduction in time for sample preparation, as threshing is not required for model attainment of grain numbers. Given the commonly two-rowed physiology of the barley grain head relative to other major crops such as wheat, rice, and oat, the development of a model capable of grain detection with reduced sample processing is more plausible. This is due to the reduced risk of hidden and/or clustering grains that avoid detection during image processing [45,46,47,48]. In addition, we highlight the simplistic nature of the image capture protocol (merely requiring a standard computer, a camera, simple tripod and black card), thus making BarleyGC an accessible and cost-effective choice for researchers who may have difficulty securing more sophisticated sensor-based phenotyping equipment [49]. However, we also highlight the limitations of our model for direct counting of grains from spikes overlayed into diverse backgrounds, given our utilisation of black-background only images. We additionally note potential limitations of the model for deployment on six-rowed barley heads, where grains would likely be obscured, thus hindering model-based grain detection. This would effectively limit the scope of BarleyGC applications in scenarios where grain detection includes six-rowed barley phenotypes, which primarily encompasses the germplasm of varieties in the feed export trade [50,51]. In light of this, there may still be a potential applicability of our pipeline to 6-rowed barley. A study by Qiu et al., (2022) [52] successfully implemented a grain detection pipeline utilising deep convolutional neural network (DCNN) architecture in wheat. Wheat exhibits spikelet architecture with grain clustering and obscuring of grains in a single image, similar to six-rowed barley [52]. The authors overcame this by capturing a second image of each sample, after a 180 degree rotation [52]. In turn, the grain numbers from image pairs could be effectively combined post-processing.

As highlighted, BarleyGC demonstrated highly satisfactory capacity for grain quantification showing exceptionally concordant model scaling with phenotypic data variation, adding an average of 0.98 grains for every true grain counted during the manual phenotyping process (Figure 2A). BarleyGC was thus considered an adequate image processing pipeline, without the need for manual correction of model-generated data (given observed scaling behaviour). BarleyGC performance is promising—especially given its training on a relatively small dataset (1000 images), and limited genotype pool of DH barley lines (all derived from the same parental varieties). Yet, we note the potential for bias that may arise from accuracy validation of an image training pipeline used to phenotype the same genetic population that was used to complete training. Wu et al. (2025) note these caveats in a separate study by Fernandez-Gallego et al., (2018), which captured top-down wheat and barley images via a simple camera/tripod approach [53,54]. Despite high accuracy of the model used to train on the 25 genotypes under the specific field conditions, Wu et al. (2025) highlighted that morphological variability in untested genotypes, weather and/or the presence of weeds as potential issues during real-world deployment [53]. Due to these constraints on model accuracy, such caveats may be improved with the incorporation of additional variables to future training runs. Such improvements include the introduction of a substantially expanded image training set consisting of barley head images with greater background diversity, and the use of optimised augmentation parameters (i.e., scaling, flipping, brightness and noise adjustments) [43]. Introducing varied image resolutions during successive training runs, via multi-scale training, would be thoughtful to improve model robustness on compressed images. Such compression is typical for practical image storage and transfer scenarios involving expansive sample numbers collected from the field [55,56]. We may also further improve the accuracy metrics and/or robustness for BarleyGC reported here via transfer learning. This would involve using a new training set of unprocessed sample images. These images contain the original black card backgrounds, which exhibited lighting variability from being captured at different times of day, and small sampling artefacts including small pieces of foreign plant material. In the event that model robustness is established, this would eliminate the requirement for stringent pre-processing upstream of model deployment on phenotype images. Finally, increasing the genetic diversity of barley head samples (such as the inclusion of differing grain colour phenotypes, and those with varying degrees of spikelet density/differing awn types) may allow for enhanced prediction capabilities [57,58,59].

### 3.3. Exploring the Effect of RGT Planet on Enhanced Grain Number in the DH Population as a Known Deficiens Phenotype

Parental varieties of our generated DH population (RGT Planet and Hindmarsh) both possess the two-row phenotype. This is the known ancestral configuration of the inflorescence in wild progenitors of *H. vulgare*, and subsequent ancient, domesticated lines [26,60]. Early in barley’s domestication history, derivative six-rowed phenotypes were selected for the Middle East, based on their capacity to generate three times as much grain as their two-rowed ancestors [61]. The six-row type cultivar subsequently dominated much of the domestic pool for millennia, until two-row types reappeared approximately three and a half thousand years ago [62]. These novel two-rowed lines descended from the very same six-rowed germplasm (rather than the original two-rowed progenitors), based on recent genetic and combined archaeological evidence [62]. Multiple independent instances of six-rowed type barley have been observed during *H. vulgare*’s domestication history, with *Vrs1* as the primary locus directing observed shifts in spikelet morphology [26,63].

In this study, we identified the qGN-2H QTL region as significantly associated with the grain number trait in our DH population. qGN-2H exhibited an effect size of 1.18 grains per spike, explaining 7.9% of the phenotypic variance observed. The effect size reported for qGN-2H here, is comparable with QTLs identified near the *Vrs1* region reported in other studies—such as ‘SCRI_RS_165473’—to which the favourable allele improved grain number by 2.5 grains per spike [64]. Thus, the observed consistency of small-to-moderate effect QTLs being identified near *Vrs1* in independent trials, somewhat bolsters the likelihood of qGN-2H having true biological influence on grain number variability. However, we highlight the need for additional validation of the qGN-2H locus prior to breeding programme incorporation, given our study did not validate QTL recurrence across multiple environments and time-points [65]. In addition, we must be conservative in our reporting of effect size and its impact on yield outcomes in a commercial context, as we did not measure additional traits underlying yield performance that may confound the QTL effect—in particular, grain size/weight, which are generally negatively correlated with grain number [66]. For example, studies have shown that although increasing expression of *TaBG1* and *TaCYP78A5* improved grain weight, yield was substantially impacted by a concordant decrease in grain number in wheat [67,68]. We emphasise that the QTL effect is derived from a single field trial, representing data collected over a single day, which further impounds on the statistical relevance of the reported QTL effect, compared to those reported from expansive, multi-year, multi-environment trials [69]. Finally, the QTL region was isolated from phenotypic data with low broad sense heritability (H^2^ = 0.12%), low repeatability (−0.222) and high residual variance estimated by LMM (84.7%). These characteristics define a phenotypic distribution dominated by stochastic, plot-level variation, to which detection of a low QTL signal (LOD = 3.14) was the consequence. Such large contributions of environmental effects on phenotype variability, combined with small sample size (*n* = 153 DH lines), typically restrict detection power to major-effect loci, which would otherwise exhibit substantially higher LOD scores under conditions where stochastic variation is better controlled [70]. This is reflected in our discovery of a single significant locus containing *Vrs1* in this study. Future analysis incorporating multi-environment trials are thus required to validate qGN-2H robustness and applicability in the context of yield improvement.

*Vrs1* was identified as the candidate gene linked to our grain number QTL qGN-2H, given the gene’s extensive characterisation as a major regulator of grain formation [26,63,71]. *Vrs1* encodes a homeodomain leucine zipper class 1 transcription factor [26]. Its function has been experimentally validated as a negative regulator of lateral spikelet development, with its expression isolated to the lateral florets [72]. Wildtype *Vrs1* produces the two-row, ancestral phenotype in barley. Over 50 mutations in *Vrs1* have been cumulatively documented, resulting in a range of spike phenotypes [54]. Induced reduction in *Vrs1* activity increases grain number via the formation of fertile lateral spikelets, leading to the generation of 6-row phenotypes [73]. Given the concordant increase in grain number resulting from 6-row morphology, loss-of-function *Vrs1* mutants dominated agricultural landscapes early in barley’s domestication approximately 9000 years ago [74].

Allelic segregation analysis in the DH population, revealed that the favourable allele ‘A’ at flanking marker m575 of the gGN-2H locus (imparting an extra 1.18 grains per spike), was derived from the RGT planet parent. This was somewhat unusual, given RGT Planet is known to exhibit *deficiens* morphology with extremely diminished lateral spikelet formation, leading to the development of larger grains [75]. Even more intriguing is the underlying cause of the *deficiens* phenotype in RGT planet and other affected varieties (including HOR13954)—this being a mutation in the *Vrs1* coding region [75]. The allele, termed *Vrs1.t1*, causes an amino acid conversion in exon 3, changing the native serine residue at a predicted site of phosphorylation in the C-terminal region, in turn altering *Vrs1* activity and severely reducing the lateral spikelets [72,75]. *Deficiens* varieties such as RGT planet typically exhibit superior yield stability/increased grain size (as a result of reduced resource allocation to the lateral spikelet). Our observations of enhanced grain number in DH lines with the derivative RGT Planet allele in our study, somewhat contrast with what is typically understood about *deficiens* varieties possessing the *Vrs1.t1* mutation. As outlined above, *Vrs1* is a well-known suppressor of grain development, intrinsically acting in lateral spikelet primordia [26]. Sakuma et al. (2017) [72] hypothesised that the lack of serine residue at the *Vrs1.t1* locus resulted in a subsequent reduction in overall phosphorylation. This led to persistence of transcriptional activity of *Vrs1*, and further reduction in lateral spikelet development than typically observed for genotypes with the wildtype *Vrs1* sequence. The authors also noted that the level of *Vrs1* expression (quantified by measured mRNA) was significantly lower in *deficiens* versus wildtype lines after the lemma primordium stage of floral development [72]. However, the authors postulated this was unlikely to have influenced formation of the *deficiens* phenotype, with reduced overall *Vrs1* expression being a likely artefact of severely arrested lateral spikelet development, and thus smaller overall size of the lateral spikelet, versus the wildtype [72].

Revisiting our observation of reduced *Vrs1* inflorescence expression in RGT Planet versus other barley varieties in the pangenome, we originally hypothesised a link between low *Vrs1* transcript abundance in RGT Planet and enhanced capacity for increased grain number. Given the conclusions of Sakuma et al. (2017) [72] following *Vrs1* expression analysis in deficiens variety Def2, (sharing the same mutation as RGT Planet), there is a potential that the reduced lateral spikelet dimensions (and subsequent reduction in transcript levels) may have a secondary impact on grain number. Further investigation is required to determine whether the reduced floral *Vrs1* expression in RGT planet is a direct result of the *deficiens* phenotype, or if *Vrs1* downregulation in RGT planet is due to other factors. In addition, future experiments are needed to determine whether the observed increase in grain number for DH lines carrying the favourable planet allele is due to reduced *Vrs1* transcript abundance. Such an example of external sequence factors impacting *Vrs1* expression, was shown in accessions from the Tibetan plateau carrying the 6-row allele *vrs1.a4* (with identical sequence to the two-rowed genotype *Vrs1.b4*). The lines showed reduced *Vrs1* expression due to a TA deletion 1 Kb upstream of the coding sequence, leading to the formation of a six-rowed morphology [76]. Indeed, there may be additional factors regarding regulation of *Vrs1* expression not yet identified in RGT Planet, that may help contribute to improved grain number. Future work may involve investigative measures to further assess the *Vrs1* regulation network in RGT Planet, such as through combined gene expression/transcriptomic analysis [77,78]. However, we must highlight the speculative nature of the above hypotheses surrounding the impacts of allele ‘A’ at m575 in relation to *Vrs1* functionality—especially given their grounding on a single quantitative trait locus spanning over 2.3 Mbp. As indicated in Table 3, this expansive locus contains numerous genes, and thus we cannot confidently assert that the QTL marker allele is linked to *Vrs1* without sufficient experimental evidence and/or further marker enrichment.

## 4. Materials and Methods

### 4.1. Preparation of Plant Materials and Field Conditions for the RGT Planet X Hindmarsh Doubled Haploid Population

The generation of doubled haploid progeny first involved crossing the Australian feed variety Hindmarsh, and the European elite malting variety, RGT Planet. DH development was completed by the Department of Primary Industries and Regional Development (DPIRD), using anther culture protocol adapted from Broughton et al., (2024) [35]. The resulting seeds derived from mature DH progeny were sown at the Department of Primary Industries and Regional Development (DPIRD) Research Station located in Wongan Hills, Western Australia (Geographic coordinates: Latitude −30°53′36.46″ S; Longitude, 116°43′2.87″ E). Seeds were sown in June 2023, and heads were sampled in late September 2023. The field trial encompassed a partially replicated design, with a replication rate of 22.4% and 41 of 183 DH lines being replicated. Partial replication was implemented to assist in reducing spurious associations derived from differing field composition across plots. DH lines were grown under an average solar exposure of 13.75 MJ/m^2^ over the period from June to September and were exclusively supplied by seasonal rainfall [79]. From the period of June to September of the sowing year, DH lines received an average monthly total of 33.25 mm of rainfall [79].

### 4.2. Sampling of Field Data and Image Capture Protocol

Sampling of barley heads was completed over a single day on 26 September 2023. Heads were collected by cutting approximately 1 cm below the base of the head. In total, 6–8 heads were sampled from each plot, ensuring that only plants in the middle of the two centre rows of the plot were sampled, to avoid the impacts of edge effect for plants grown in surrounding guard rows. These adjacent plants may have received greater nutrient and/or sunlight supplementation compared to their central neighbours. Imaging of collected heads from the DH population was completed with an iPhone 15 pro max, mounted to a tripod at a height of 715 mm from the ground. Individual barley heads were imaged on a black plastic surface. A paper label, with the corresponding plot number of the barley head sample, was placed in the bottom right corner, adjacent to a 150 mm ruler. Barley heads were imaged on a dark background to increase contrast between the background and grain head, for enhanced training efficiency during downstream model development. Each grain head was captured 3 times, being moved on a different angle with each photograph, to increase complexity of barley head sample presentation for enhanced accuracy of grain detection during model training. Following image capture, a total of 245 samples were photographed, comprising a total pool of 5894 barley head images.

### 4.3. Training of Grain Detection Model and Compilation of Phenotype Data

**Figure 7:**
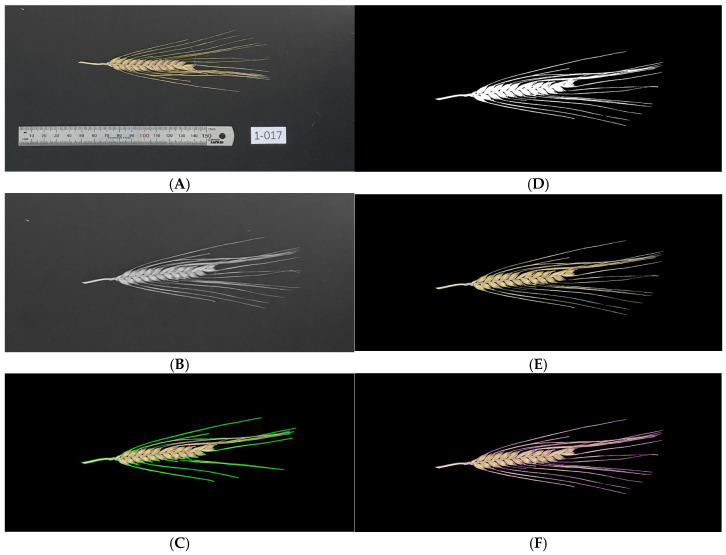
Illustrated workflow for image preprocessing with OpenCV. The workflow begins with the unprocessed raw sample image (**A**), which was then subjected to cropping and noise reduction via greyscale conversion and implementation of gaussian blur (**B**). Next, a series of refinement techniques, i.e., Otsu’s thresholding, hull dilation and contouring (**C**) led to production of binary masks (**D**), which were then overlayed onto the original cropped image to remove artefacts and maximise background contrast (**E**). Canny edge detection was also utilised for enhanced grain division during the annotation step (**F**).

Prior to training the grain detection model, the barley head image collection (*n* = 5894) was first subjected to a series of preprocessing steps, as shown in Figure 7. Preprocessing of raw images was achieved using a custom python script adapting the Open Computer Vision Library (OpenCV) version 4.11.0. The Claude Opus 4 large language model (LLM) was used to assist with code formulation and troubleshooting during script development. The preprocessing script served to reduce image noise for improved efficiency of training in the subsequent model development steps. First, grain head images were cropped to remove image labels and colours were converted to the HSV (Hue, Saturation, Value) colour space for improved contrast of the grain head and background (via colour thresholding). The result was a generated colour mask based on grain head hue values (in this case, yellow-brown upper and lower bounds were selected via the cv2.inRange function). The second mask was determined utilising the value channel to capture additional regions of the grain head that may have been previously missed, and this mask was then combined with the colour mask. Next, images were converted to grey scale and noise reduced via gaussian blur to limit the likelihood of background artefact retention in the proceeding binary mask production (Figure 7B). Ostu’s threshold was then implemented using image-specific histograms (comparing pixel count to intensity) to determine optimum threshold values for effective separation of barley heads from the background. The binary masks were then further refined with a small (2 × 2) kernal matrix. Refinement allowed for the breaking or weaker bridges that may have linked artefacts present in the background to the barley head object. The pipeline then identified regions for contouring stored in the temporary binary and found the largest contour (predicted to be the barley head object). Gaps in the contour were then closed with ConvexHull and the hull dilated (via a larger 15 × 15 kernal matrix) for enhanced contour detail for barley head capturing (Figure 7C). The previous result of Ostu’s thresholding was then combined with the dilated hull through the cv2.bitwise_and operation, leading to the creation of the final_binary mask object (Figure 7D). The final_binary mask was then fitted to the original image, only capturing the image region inside the generated mask area. This effectively led to retainment of just the barley heads in the images and removal of all background areas, with these being replaced by a high contrast black background (Figure 7E). The final step of image preprocessing involved implementation of canny edge detection (following greyscale conversion and bilateral filtering) to generate coloured contours for improved separation of grains during manual annotation (prior to image training) (Figure 7F). Images without the edge detection (final binary mask only) were used for actual model generation, due to edge colours not being representative of true sample appearance.

Following the preprocessing step, 1000 randomly selected images from the barley head collection, consisting of varied spike presentations (i.e., angle and positioning in the image frame), were annotated using COCO software (version 0.11.0). Annotations for individual grains were generated manually with the polygon tool. The completed annotated dataset consisted of 25,005 instances (labelled objects) for the ‘grain’ class—over twice the recommended per class for object detection, as per the relevant documentation for Ultralytic’s You Only Look Once (YOLO) framework (https://docs.ultralytics.com/yolov5/tutorials/tips_for_best_training_results/; accessed on 2 May 2025). The completed annotations for the 1000 images in the training set were then exported in ‘.json’ format. Prior to training, COCO-formatted annotations were converted to YOLO format, using a custom script that scanned the directory containing annotated images, matching image names to those located in the annotation file to generate a newly formatted set of annotations that can be interpreted for training. Training was completed using Ultralytic’s You Only Look Once (YOLO) version 11 framework (https://docs.ultralytics.com/tasks/detect/#train; accessed on 12 May 2025), deriving pre-trained weights from the Microsoft COCO: Common Objects in Context 2017 dataset—comprising 2.5 million instances spanning across over 80 object categories, and >300 thousand images [80]. For enhanced accuracy, the large model (yolo11L.pt) was selected for training. The image set was first split into separate train and validation directories, consisting of 801 and 201 images respectively, to reduce the likelihood of negative outcomes—in particular, overfitting of the model to the provided images (which would lead to abnormal performance on future new image sets). ‘No sample’ empty image and label files were included in the relevant training and validation directories, to ensure model capacity to correctly interpret cases of missing data. The .yaml configuration file contained the location of training and validation datasets, in addition to specifying the number of classes (nc = 1 for the detection of barley grains only), and annotated labels. Parameters specified a detection model with a training run of 400 epochs, a batch size of 4 (to accommodate the computational demand of the large YOLO model). AMP (automatic mixed precision) was used to reduce memory usage and improve training speed via interchangeably utilising both half- (16 bit floating point) and full-precision (32-bit floating point) memory formats. Training was performed on hardware possessing the NVIDIA GeForce RTX 3060 Ti graphics card (manufactured by Nvidia Corporation and sourced from Perth, Australia), with 8 GB of VRAM and CUDA software version 12.6. An additional 200 epoch training run was completed, with a reduced batch size of 1 and increased image resolution (832 × 832 pixels) for enhanced image preservation to further improve model accuracy.

The finalised model (BarleyGC) was then used in combination with the segment anything 2 (SAM2) model (the large model sam2.1, utilising Hiera architecture), to detect grains and generate segmentation masks for individual grains, respectively (https://docs.ultralytics.com/models/sam-2/#how-to-auto-annotate-with-sam-2; accessed on 12 May 2025). These tasks were completed with an auto-annotation pipeline that generated new annotations with the aforementioned models (in COCO’s .json format) for provided barley head images. Claude Opus (versions 4 and 4.1) was utilised to aid in pipeline curation, assisting during the coding/debugging steps. For increased capacity for grain detection (and reduced likelihood for missing true grains in processed images), the confidence threshold for the train4 model was set to a value of 0.2, whereas the SAM2 confidence threshold was set to a more conservative 0.7. This was done to both reduce abnormal grain detections and ensure accurate formation of segmentation masks for detected grains. Phenotype data was prepared from the original barley head image collection by manually selecting 3–7 unique images (averaging 4 per genotype), representing the technical replicates for each genotype (field plot). Following phenotype data organisation, a total of 973 images (comprising 245 lines) were processed with the custom pipeline, and the generated annotations then cleaned with COCO to remove any artefacts and abnormal grain detections to obtain the final phenotype dataset.

Accuracy metrics for model- and manually derived grain numbers per spike, were calculated in base R using data from the manual phenotyping of the barley head image collection (*n* = 973) and model-predicted data generated using the auto-annotation pipeline (combining both the BarleyGC detection model and SAM2 model). Pearson correlation and linear regression analysis was completed on manual and model counts. Errors were calculated by subtracting the manual count data from the model count data, with the mean error used in the calculation of mean error bias, and mean absolute error (MAE). A frequency histogram of error distribution, in addition to the linear regression model with plotted model and manually derived grain number measurements, was generated with ggplot2 (version 4.0.1). 2D-kernel density estimation was applied to the scatterplot. This was done to better visualise the frequency of datapoints displaying a high degree of correlation between the model- and manual- derived grain detections. Opacity was determined based on local-point density, as calculated over a 100 × 100 grid range, using the kde2d() function included in the MASS package (version 7.3-60.2). Datapoints were assigned the density value (opacity level) of the nearest cell in the grid, with darker opacities corresponding to higher density classifications. Random jitter (amount = 0.3) was applied to the scatterplot, to improve visualisation and counteract overplotting that occurred as a result of mapping hundreds of discrete data points.

### 4.4. Investigation of Phenotype Data Prior to QTL Analysis

Phenotype data was collected for 245 plot samples, consisting of a combination of DH lines (*n* = 232 DH lines) and non-DH commercial varieties, including the RGT Planet and Hindmarsh parent lines (*n* = 13). Phenotype data consisted of average grain number per spike values, derived from the technical replicates of each plot (*n* ≈ 4, with a range of 3–7). After removing DH lines missing a unique ‘DArT genotype’ file identifier, there was a total of 229 DH line plots remaining. Accounting for the partial replication trial design (covered in Section 4.1), 183 unique DH lines were available for downstream QTL detection. Of the 41 DH lines with replicated plots, 36 were two-plot replicates, and 5 were three-plot replicates. The average coefficient of variation (CV) values for both two- and three- plot genotypes, were calculated in RStudio (version 2024.04.2), using the dplyr package (version 1.1.4). Next, the cor() function was used to calculate the Pearson correlation coefficient between the 36 duplicate plot values (this being the average number of grains per spike for each plot pair), thus determining repeatability of duplicated plot measurements. Variance partitioning was completed with the plot values for grain number per spike (*n* = 229), in R, using the lme4 (version 1.1-37) and lmerTest packages (version 3.1-3) implementing the simple row column mixed linear model. The linear mixed model (LMM) incorporated variables representing random effects for both genotype (*g*) and spatial (range/row) coordinates (*r* and *c*), with the intercept (population mean grain number per spike), as the fixed effect (µ). The equation for the simple mixed linear model is adapted from Neupane Adhikari et al., (2016) [81] and is defined below. The model comprises the various components defined above, plus the residual effect (ε) determining the value of any given plots ‘grain number per spike’ measurement, in an additive fashion [81].

*y* = μ + *g* + *r* + *c* + ε

Variance components were additionally estimated, using a mixed linear model fit by REML (Restricted Maximum Likelihood), for an unbiased approach accounting for degrees of freedom potentially lost due to fixed effects. The plot-level broad-sense heritability was calculated using the derived variance components, collectively representing the total phenotypic variance ($\sigma_{P}^{2}$). Heritability was calculated via the following equation below, where $\sigma_{G}^{2}$, $\sigma_{R}^{2}$, $\sigma_{C}^{2}$ and $\sigma_{\varepsilon }^{2}$ represent the variance component for genotype, row, range and residual variance respectively;

$$H^{2}=\frac{\sigma_{G}^{2}}{\sigma_{G}^{2}+\sigma_{R}^{2}+\sigma_{C}^{2}+\sigma_{\varepsilon }^{2}}$$

Finally, phenotype data was prepared for QTL analysis by deriving a single representative value for grain number per genotype. This was achieved by calculating simple means for grain number between biological replicates for multi-plot genotypes. For outlier genotypes (*n* = 8) that were determined to potentially negatively impact the statistical power of subsequent analysis (with a difference of >5 grains between replicate plots), the median was instead used before proceeding to QTL identification. For triplicate plots, the median was used if any two replicates differed by more than 5 grains. Outlier classification for replicates was determined based on assessing the distribution of absolute differences between replicate plots. Plotting the absolute difference revealed distinct separation between the main distribution (where majority of values clustered) and the tail (consisting of what were considered outlier values) at 5 grains—thus making the value desirable as an outlier threshold (Supplementary File, Figure S3). The 5 grain value equated to 1.69 times the within genotype SD (2.96), and 1.81 times the between genotype SD (2.76), which despite being below the typical 2 standard deviation threshold for outlier classification, allowed for substantial control of plot-level stochastic variation while reducing the risk of removing true biological variation in the phenotype data [82]. Following correction for outliers, the median-adjusted data was assessed for normality. This was done through combined assessment of a frequency histogram plotting average grain number per spike values across the 183 genotypes, and Shaprio–Wilk test. The Shaprio–Wilk test was performed using the shapiro.test() function in RStudio, indicating normality at *p* > 0.05. Finally, Repeatability was performed a second time following removal of outliers from duplicate plot values (*n* = 30 plots), using the Pearson correlation method outlined above.

### 4.5. Identification of Quantitative Trait Loci for the Grain Number Trait and Statistical Analysis of QTL Marker(s)

Prior to the QTL scan, marker data for the DH population was processed and formatted for use with the R package qtl2 (version 0.38). The genetic map data was generated in 2019, using DArT (Diversity Arrays Technology, Canberra, Australia) sequencing protocol (https://www.diversityarrays.com/services/dartseq/; accessed on 12 January 2026). The MorexV1 assembly was used as the reference genome (genome build accession: GCA_901482405.1) [83]. First, DH line IDs were matched between the phenotype and genotype data files, leading to the retainment of 153 genotypes with matching IDs between both files. The 153 matching IDs represented DH lines with genotype data present in the map file and thus formed the finalised mapping population for QTL detection. The genetic map data comprised an initial 3357 total markers, which were filtered to exclude both markers on ChrUn and not assigned to a chromosome, leaving 2776 total markers for QTL analysis (Table 4). DH lines could possess either the RGT Planet-derived allele ‘A’ or the Hindmarsh-derived allele ‘B’ at a given marker position. The mean A allele frequency was 0.524, and no markers in the genetic map exhibited nearly fixed allele distributions (i.e., >90% of the same allele A or B). Using the genotype file and genetic map file (containing chromosome positions for the respective markers) genotype probabilities were calculated for the DH population across all markers, with a defined error probability of 0.002. For lines with missing genotype data at a particular marker, flanking markers were used to estimate genotype probability. The genotype probability matrix (in conjunction with the phenotype data), was then used to perform the initial QTL scan. The LOD scores at each marker were determined following calculation of the residual sum of squares (RSS) values, derived from two linear models—the ‘null’ model, which does not incorporate the QTL effect, and the ‘full’ model, which incorporates the QTL effect. Thus, LOD scores were calculated by comparing the RSS values from both the null and full linear models, with the following equation below (where ‘n’ is the sample size):

LOD = (n/2) × log_10_(RSS_null/RSS_full)

Following the initial QTL scan, the average kinship (proportion of alleles in common between any two DH lines across all markers) was calculated with the genomic relationship matrix (GRM) method. The GRM method determined the extent of relatedness between DH lines, presented as pairwise kinship similarity scores, and calculated from the original genotype probability matrix. Following assessment of kinship, two additional QTL scans were performed, using a kinship-based approach, and the results of principle component analysis on the DH population. We first implemented a mixed linear model applying kinship as the random polygenic effect accounting for relatedness, via matrices generated using the leave-one-chromosome-out (LOCO) method [84]. The LOCO method utilises kinship matrix values for all chromosomes, except the one to which the marker being tested resides, in turn, protecting against proximal contamination [85,86]. Principle component analysis was performed using the prcomp() function in R with both centring and scaling, using 2756 filtered markers (removing markers with ≥20% missing data). The first five principal components (PCs) were used as fixed-effect covariates in the revised QTL scan. Clustering analysis was additionally performed, using the k-means algorithm on the first 3 PCs for improved visualisation of population structure. Finally, ANOVA (nested model F-test) was used to compare two linear models—model 1 (the base model for grain number prediction with cluster effect) and model 2—accounting for both cluster effects and the additional QTL effect (assuming equal QTL effect across clusters), to determine if population structure was confounding the QTL signal.

Prior to completion of the above outlined QTL scan analysis, permutation testing was carried out to derive the LOD threshold for statistical significance at α = 0.05. Permutation tests were completed with qtl2, using the scan1perm() function for 1000 permutations. To enable reproducibility of the results, a fixed random seed was defined (set.seed(123)). For the kinship-corrected and PC-corrected permutation tests, the same data was utilised for completion of the QTL scan, these being the LOCO-derived matrices and first 5PC values for each, respectively.

Allelic segregation was investigated at the marker m575, located at the significant LOD peak. The mean grain number for allele A (*n* = 92) and allele B (*n* = 57) was calculated and a Student’s *t*-test performed to compare the distribution of grain number measurements at both alleles. In the event of a statistically significant difference in grain number between alleles, the QTL effect size was determined by the difference in means between the A and B allele. The phenotypic variance explained (PVE) at marker m575 was then determined via a linear model comparing the correlation between grain number and genotype in the DH population at the marker position. PVE was determined by R^2^ (derived from the linear model) × 100.

### 4.6. Characterisation of Candidate Gene(s) Within the QTL Interval

The QTL region qGN-2H at 224.959 cM, defined by flanking markers m574 and m575, was investigated to identify candidate genes for grain number in the DH population. The qGN-2H QTL interval on chromosome 2H, spanning 651,160,368–653,534,354 bp, was searched using annotation data for the Morex V1 genome assembly, retrieved from the Ensembl Plants database (http://ftp.ensemblgenomes.org/pub/plants/release-51/gff3/hordeum_vulgare/; accessed on 26 November 2025). The protein sequences for the candidate genes were additionally retrieved from Ensembl (http://ftp.ensemblgenomes.org/pub/plants/release-51/fasta/hordeum_vulgare/pep/Hordeum_vulgare.IBSC_v2.pep.all.fa.gz; accessed on 26 November 2025). Claude Opus 4.5 was used to assist in script generation to integrate the above processes in RStudio. Protein sequences were submitted to the NCBI CD batch search tool https://www.ncbi.nlm.nih.gov/Structure/bwrpsb/bwrpsb.cgi; accessed on 26 November 2025) to infer potential gene functions based on domain architecture. Literature searches assisted in the subsequent prediction of gene function, using primary domain architecture of candidate genes identified in the QTL interval. For a subset of candidate genes of interest (namely HORVU2Hr1G092460 and HORVU2Hr1G092290), the UniProt BLAST function (https://www.uniprot.org/blast; accessed on 27 November 2025) was used to identify top protein hits and subsequently identify the equivalent gene annotation from the latest MorexV3 genome assembly (https://graingenes.org/GG3/content/morex-v3-files-2021; accessed on 13 January 2026). The MorexV3 annotation were then submitted to the PanBARLEX Lift-Over tool to generate the equivalent pangenome gene ID(s), which were subsequently used to investigate the expression profile across 5 tissue states (embryonic, root, shoot, inflorescence and caryopsis). This was completed for the associated gene cluster for candidate genes HORVU2Hr1G092460 and HORVU2Hr1G092290, across the 20 pangenome accessions in the PanBARLEX interface.

## 5. Conclusions

The regulation of yield-related traits is of major importance to the global barley industry, as a means for both safeguarding food security and improving economic potential. Identification of grain number-related polymorphisms remains a prominent research endeavour, with novel avenues for accelerated phenotyping of yield-related traits offering rapid avenues for QTL discovery. In recent years, a series of image processing models have been constructed for grain number quantification in agronomically predominant crops, including wheat and rice. Here, we reported the training of a novel grain detection model in barley, with high accuracy metrics and reduced sample processing requirements. The model is capable of counting grains directly from images of intact barley heads in two-rowed varieties. Our model showed satisfactory capacity for grain detection, leading to the subsequent identification of a moderate effect QTL on chromosome 2H, defining an interval containing the candidate gene *Vrs1* (a pivotal regulator of spikelet morphology and row-number in *H. vulgare*). Overall, our study effectively demonstrates the suitability of BarleyGC for the task of grain detection and downstream QTL isolation. The results illustrate the adaptive utility of the YOLOv11 framework, thus showing promise for the development of accessible, low-cost, high-throughput phenotyping protocol for the grain number trait in barley.

## Figures and Tables

**Table 1:** Results of phenotype data analysis for the DH population used in QTL identification.

Panel (A): Coefficient of variation for plots with repeated genotypes			
Number of plot replicates	Number of genotypes	Average CV	Average range (grain number per spike)
2	36	0.084	3.01
3	5	0.107	5.06
Panel (B): Repeatability values (for the trait of average grain number per spike) across duplicate plots			
Dataset type		Repeatability	
All duplicated genotypes (n = 36)		−0.222	
Duplicated genotypes with outliers removed (n = 30)		0.397	
Panel (C): Variance components for the DH population (n = 229 plots)			
Effect	Variance component		Percentage (%)
Genotype	0.009938871		0.1
Spatial (Row)	0.786657886		9.1
Spatial (Range)	0.523202051		6.1
Residual	7.286333883		84.7

CV, coefficient of variation. Statistical values were derived from mean grain number per spike measurements, calculated from the technical replicates from each plot (*n* ≈ 4 heads per plot, range 3–7). Repeatability was determined using Pearson correlation between mean grain number per spike measurements for duplicated genotype plots. Outliers were defined as differing by more than 1.81 standard deviations (>5 grains) from the mean grain number per spike for the DH population (consisting of 183 unique lines across 229 plots). Variance components were calculated using a simple row column Mixed Linear Model with row, range and genotype defined as random effects, fitted by Restricted Maximum Likelihood (REML). All analyses were completed in RStudio (version 2024.04.2) using lme4 (version 1.1-37) lmerTest (version 3.1-3) and dplyr (version 1.1.4) packages.

**Table 2:** Results of ANOVA (nested model F-test), which compared two linear models—grain number prediction via cluster assignment only, and with the additional effect of QTL qGN-2H (defined by marker m575 on the physical map). Additional parameters (degrees of freedom) added to the respective models are shown (df), with their residuals (145). Also included are the F-statistic for the respective linear models, and their associated *p*-values. Significance level is denoted as *p* < 0.001 (***).

Model	df	F-Value	*p*-Value
Cluster only	2, 145	1.17	0.314
Cluster + QTL (qGN-2H, marker m575)	1, 145	11.41	0.00094 ***

**Table 3:** Candidate genes identified with domain hits in the QTL interval defined by qGN-2H, spanning 2.37 Mb (651,160,368–653,534,354 bp) on Chromosome 2 in the MorexV1 genome assembly (Genome build: GCA_901482405.1). The gene ID, gene start position (Mb), primary domain feature and gene type classification are provided below. Gene function was determined through a combination of conserved domain search (CD-Search), UniProt protein BLAST (release 2025_04) functions and literature searches based on primary domain architecture.

Gene ID	Position (Mb) chr2H	Primary Domain Feature	Associated Function	Reference(s)
HORVU2Hr1G092170	651.248931	Unknown	Unknown	-
HORVU2Hr1G092180	651.370368	EF-G (Elongation Factor G)	Protein translation	[19]
HORVU2Hr1G092190	651.377631	TPR (Tetratricopeptide repeat)	Diverse functions	[20]
HORVU2Hr1G092200	651.398906	Non-Translating CDS	Unknown	-
HORVU2Hr1G092230	651.461031	ATG13 (Autophagy)	Stress response	[21]
HORVU2Hr1G092250	651.528086	DUF3490/Kinesin	Growth regulation	[22]
HORVU2Hr1G092260	651.534346	BRCT (DNA repair)	Stress response	[23]
HORVU2Hr1G092270	651.676342	VOC/Glyoxalase	Stress response	[24]
HORVU2Hr1G092280	651.692336	NADH dehydrogenase	Respiration	[25]
HORVU2Hr1G092290	652.031058	Homeodomain (HOX)	Transcription factor	[26]
HORVU2Hr1G092300	652.093963	Homeodomain (partial)	Transcription factor	[26]
HORVU2Hr1G092340	652.416364	Unknown	Unknown	-
HORVU2Hr1G092350	652.463088	WD40 repeat	Diverse functions	[27]
HORVU2Hr1G092360	652.499287	Cytochrome P450	Diverse functions	[28]
HORVU2Hr1G092370	652.710524	Unknown	Unknown	-
HORVU2Hr1G092380	652.744663	JmjC (Histone demethylase)	Chromatin modification	[29]
HORVU2Hr1G092390	652.762994	PGK2 (Phosphoglycerate kinase)	Metabolism (Glycolysis/Calvin cycle)	[30]
HORVU2Hr1G092400	652.765009	Unknown	Unknown	-
HORVU2Hr1G092420	653.323469	Unknown	Unknown	-
HORVU2Hr1G092430	653.414730	BAR domain	Sub-cellular kinetics	[31]
HORVU2Hr1G092460	653.533189	B-box zinc finger	Transcription factor	[32,33]

**Table 4:** Marker distribution across chromosomes in the *H. vulgare* genetic map used in QTL mapping analysis. The map was derived from a DH population resulting from a cross of Hindmarsh and RGT planet barley varieties.

Chr1H	Chr2H	Chr3H	Chr4H	Chr5H	Chr6H	Chr7H	Total
204	493	472	236	609	308	454	2776

## Data Availability

Data supporting the conclusions presented can be found within the article and Supplementary Material. Further enquiries may be directed to the corresponding author.

## Supplementary Materials

The following supporting information can be downloaded at: https://www.mdpi.com/article/10.3390/plants15101518/s1, Figure S1: QTL scan results following the standard scan without correction (A), scan corrected using principal component analysis (PCA) with the first five PCs as covariates (B) and utilizing kinship-based correction (via the leave one chromosome out (LOCO)) method in plot (C). The minimum logarithm of the odds (LOD) score for statistical significance was determined via permutation testing, with 1000 permutations at a genome-wide significance threshold of α = 0.05. The permutation-derived LOD threshold for significance is plotted above (red-dashed line) and the peak LOD score is plotted in green; Figure S2: Screenshot of the gene expression profile of Candidate gene HORVU2Hr1G092290/HORVU.MOREX.r3.2HG0184740 (A) and HORVU2Hr1G092460/HORVU.MOREX.r3.2HG0184930 (B), for 20 barley pangenome accessions taken from the PanBARLEX database (https://panbarlex.ipk-gatersleben.de/; accessed 20 November 2025). Expression was measured for five tissue types (Embryo, Root, Shoot, Inflorescence and Caryopsis). The expression level is provided as ‘Transcripts per Million’ (TPM), with the mean TPM provided across all genes/replicates at the bottom of each tissue category; Figure S3: Distribution of absolute difference values for the trait of ‘Average grain number per spike’ calculated for duplicated plots (*n* = 36). The outlier threshold of 5 grains is labelled below; Table S1: Statistics following QTL locus identification analysis, for three methodologies.
