# Supplementary material for: An Effective YOLOv11 Grain Detection Model Trained on Intact Barley Spikes Reveals a QTL Containing a Pivotal Regulator of Lateral Spikelet Formation
Source: Plants (Basel). 2026 May 15;15(10):1518. doi: 10.3390/plants15101518 (PMC13211034; doi:10.3390/plants15101518)

Supplementary material: "An effective Yolov11 detection model trained on in-tact spikes, reveals a QTL for grain number containing a pivotal homeodomain gene directing lateral spikelet formation in H. vulgare"

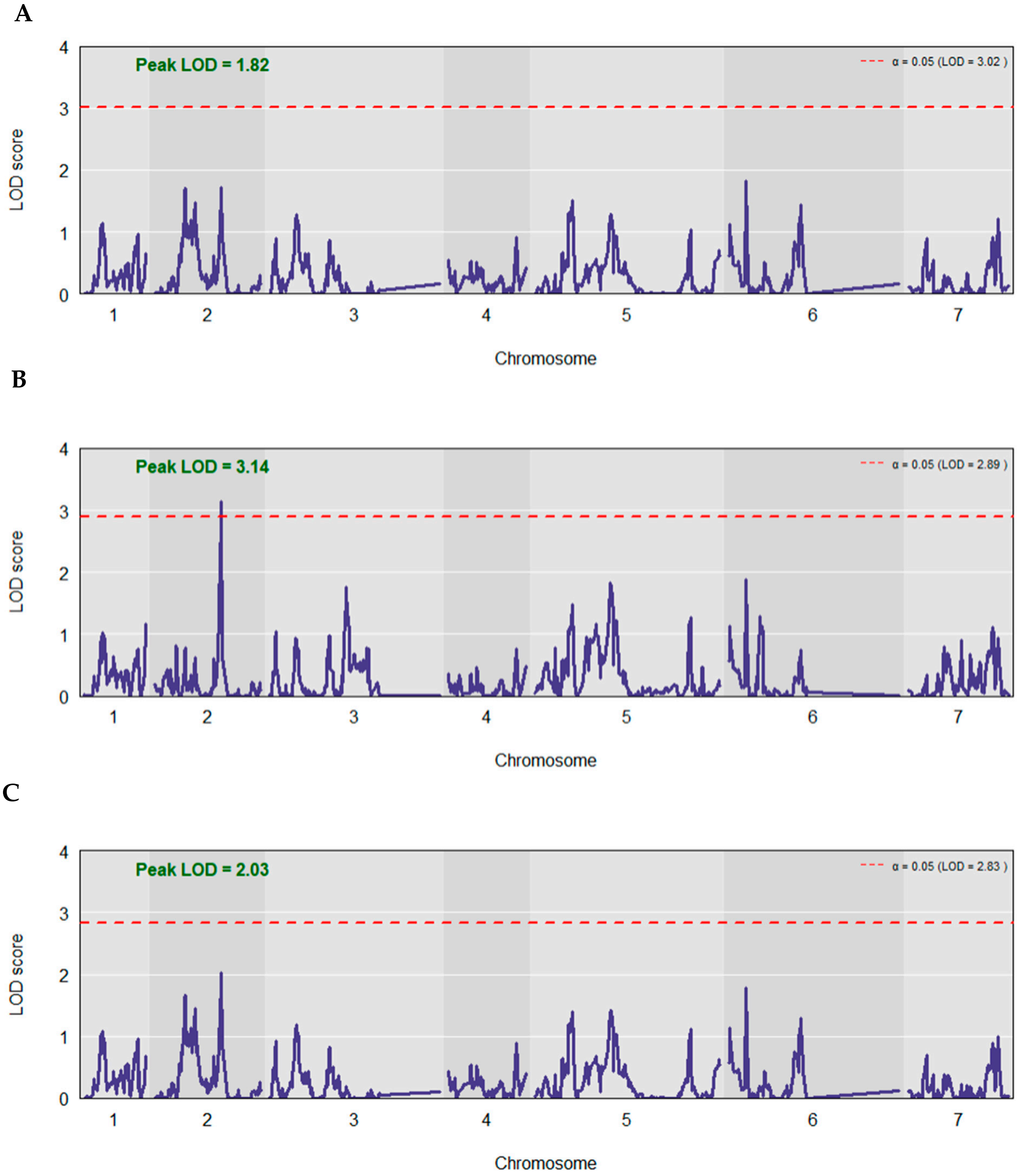

Figure S1. QTL scan results following the standard scan without correction (A), scan corrected using principal component analysis (PCA) with the first five PCs as covariates (B) and utilizing kinship-based correction (via the leave one chromosome out (LOCO)) method in plot C. The minimum logarithm of the odds (LOD) score for statistical significance was determined via permutation testing, with 1000 permutations at a genome-wide significance threshold of α = 0.05. The permutation-derived LOD threshold for significance is plotted above (red-dashed line) and the peak LOD score is plotted in green.

Table S1. Statistics following QTL locus identification analysis, for three methodologies.

**Method**	**Chromosome**	**Position (cM)**	**Peak LOD**	**Threshold (α = 0.05)**
Uncorrected	2H	224.96	1.82	3.02
PC-corrected (5 PCs)	2H	224.96	3.14	2.89
Kinship-corrected (LOCO)	2H	224.96	2.03	2.83

LOD, logarithm of the odds; PC, principal component. Kinship correction was performed using the leave-one-chromosome-out (LOCO) method, and PC-based correction was completed using the first five PCs as covariates. The LOD threshold for the respective QTL identification methods, was determined by permutation testing, using 1000 permutations for a genome-wide significance threshold of α = 0.05.

A

(Transcripts Per Million). Assembled transcripts have been mapped to the chromosome-scale assemblies of the 20 genotypes using GMAP [2]. After mapping gffcompare [3] was used to create intersections between the transcripts of the reference gene annotations and the RNA-seq-based transcripts.

Display heatmap of expression levels for: only complete, exact matches (gffcompare class code: =) — of the following transcripts: all

Gene ID (● cultivar ● landrace ● wild)	Type		Embryonic			Root			Shoot			Inflorescence			Caryopsis		
			Replicate 1	Replicate 2	Replicate 3	Replicate 1	Replicate 2	Replicate 3	Replicate 1	Replicate 2	Replicate 3	Replicate 1	Replicate 2	Replicate 3	Replicate 1	Replicate 2	Replicate 3
HORVU.AKASHINRIKI.PROJ.2HG00165680.1	cultivar	view alignment	0.00	0.00	0.00	0.34	0.00	0.25	0.03	0.00	0.00	20.30	12.01	26.73	6.10	1.86	3.04
HORVU.B1K-04-12.PROJ.2HG00166480.1	wild	view alignment	0.00	0.00	0.00	0.00	0.00	0.00	0.00	0.00	0.00	25.37	26.61	18.89	0.00	0.00	0.00
HORVU.BARKE.PROJ.2HG00167790.1	cultivar	view alignment	0.00	0.00	0.00	0.00	0.32	0.00	0.00	0.00	0.00	14.74	20.45	22.80	0.00	0.00	0.00
HORVU.BARKE.PROJ.2HG00167790.2	cultivar	view alignment	0.00	0.00	0.00	0.00	0.00	0.00	0.00	0.00	0.00	0.49	0.87	0.66	0.00	0.00	0.00
HORVU.GOLDEN_PROMISE.PROJ.2HG00164530.1	cultivar	view alignment	0.00	0.00	0.00	0.00	0.00	0.00	0.00	0.00	0.00	38.64	44.88	30.11	0.00	0.00	0.00
HORVU.HOCKETT.PROJ.2HG00164510.1	cultivar	view alignment	0.00	0.00	0.00	0.00	0.00	0.00	0.00	0.00	0.00	22.73	20.17	19.54	0.00	0.00	0.00
HORVU.HOR_10350.PROJ.2HG00159340.1	landrace	view alignment	0.00	0.00	0.00	0.00	0.00	0.00	0.00	0.00	0.00	20.82	20.24	25.41	4.18	3.68	n/a
HORVU.HOR_13821.PROJ.2HG00166150.1	landrace	view alignment	0.00	0.00	0.00	0.00	0.00	0.00	0.00	0.00	0.00	9.92	5.65	8.64	0.00	0.00	0.00
HORVU.HOR_13942.PROJ.2HG00163080.1	landrace	view alignment	0.00	0.00	0.00	0.06	0.00	0.00	0.00	0.00	0.00	31.35	32.86	28.19	11.66	7.26	9.63
HORVU.HOR_21599.PROJ.2HG00164410.1	landrace	view alignment	0.00	0.00	0.00	0.00	0.00	0.00	0.00	0.00	0.00	18.15	15.58	26.60	0.00	0.00	0.00
HORVU.HOR_3081.PROJ.2HG00163810.1	cultivar	view alignment	0.00	0.00	0.00	0.00	0.00	0.00	0.00	0.00	0.00	6.47	6.65	5.10	0.60	2.70	6.35
HORVU.HOR_3365.PROJ.2HG00163050.1	landrace	view alignment	0.00	0.00	0.00	0.00	0.00	0.00	0.00	0.00	0.00	16.27	15.28	14.19	2.80	4.08	5.99
HORVU.HOR_7552.PROJ.2HG00166240.1	landrace	view alignment	0.00	0.00	0.00	0.21	n/a	0.00	0.00	0.00	0.00	20.35	20.16	25.54	5.68	8.33	7.89
HORVU.HOR_8148.PROJ.2HG00164210.1	landrace	view alignment	0.00	0.00	0.00	0.00	0.00	0.00	0.00	0.00	0.00	3.92	13.86	n/a	0.00	0.00	0.00
HORVU.HOR_9043.PROJ.2HG00165760.1	landrace	view alignment	0.00	0.00	0.00	0.00	0.00	0.00	0.00	0.00	0.00	12.39	6.83	7.77	14.50	13.08	33.88
HORVU.IGRI.PROJ.2HG00163500.1	cultivar	view alignment	0.00	0.00	0.00	0.00	0.00	0.00	0.00	0.00	0.00	19.71	17.50	28.61	0.00	0.00	0.00
HORVU.MOREX.PROJ.2HG00162640.1	cultivar	view alignment	0.00	0.00	0.00	0.00	0.00	0.00	0.00	0.00	0.00	19.99	23.10	14.55	5.20	5.48	5.58
HORVU.OUN333.PROJ.2HG00166710.1	landrace	view alignment	0.00	0.00	0.00	0.00	0.00	0.00	0.00	0.00	0.00	4.41	5.49	2.61	2.16	0.00	2.59
HORVU.OUN333.PROJ.2HG00166710.2	landrace	view alignment	0.00	0.00	0.00	0.00	0.00	0.00	0.00	0.00	0.00	0.16	0.29	0.30	0.00	0.00	0.13
HORVU.RGT_PLANET.PROJ.2HG00163640.1	cultivar	view alignment	0.00	0.00	0.00	0.00	0.21	0.00	0.00	0.00	0.00	3.50	4.42	2.02	0.00	0.00	0.00
HORVU.ZDM01467.PROJ.2HG00163430.1	landrace	view alignment	0.00	0.00	0.00	0.57	0.63	0.32	0.00	0.00	0.00	28.68	2.99	20.17	14.42	16.27	18.69
HORVU.ZDM02064.PROJ.2HG00165700.1	landrace	view alignment	0.00	0.00	0.00	0.00	0.00	0.00	0.00	0.00	0.04	15.25	29.67	14.09	6.95	4.60	6.40
Mean TPM per tissue across all genes and replicates				*0.00*			*0.04*			*0.00*			*16.03*			*3.72*	

B

Display heatmap of expression levels for: only complete, exact matches (gffcompare class code: =) — of the following transcripts: all

Gene ID (● cultivar ● landrace ● wild)	Type		Embryonic			Root			Shoot			Inflorescence			Caryopsis		
			Replicate 1	Replicate 2	Replicate 3	Replicate 1	Replicate 2	Replicate 3	Replicate 1	Replicate 2	Replicate 3	Replicate 1	Replicate 2	Replicate 3	Replicate 1	Replicate 2	Replicate 3
HORVU.AKASHINRIKI.PROJ.2HG00165890.1	cultivar	view alignment	6.21	6.45	7.70	21.19	20.66	19.78	15.01	8.73	13.29	0.38	0.96	2.49	1.93	2.05	1.70
HORVU.AKASHINRIKI.PROJ.6HG00533490.1	cultivar	view alignment	1.45	1.73	1.48	7.12	6.37	7.89	1.60	0.86	2.10	0.35	0.97	1.01	0.19	0.32	0.17
HORVU.B1K-04-12.PROJ.2HG00166710.1	wild	view alignment	8.95	8.05	8.00	46.68	45.20	43.46	5.71	5.42	4.91	0.57	2.32	4.07	0.69	1.09	0.53
HORVU.B1K-04-12.PROJ.6HG00542730.1	wild	view alignment	3.86	3.91	4.80	1.71	1.92	2.17	0.21	0.15	0.12	1.45	1.74	2.64	0.08	0.18	0.04
HORVU.BARKE.PROJ.2HG00167970.1	cultivar	view alignment	4.58	4.19	4.15	44.45	35.87	41.26	6.36	6.02	4.98	1.09	1.06	0.82	0.75	1.07	1.59
HORVU.BARKE.PROJ.6HG00542720.1	cultivar	view alignment	1.95	1.69	1.20	18.11	16.94	12.99	2.15	2.04	1.46	1.15	2.94	0.70	0.30	0.16	0.15
HORVU.GOLDEN_PROMISE.PROJ.2HG00164730.1	cultivar	view alignment	5.46	5.09	5.69	49.77	43.74	40.75	3.08	3.83	4.20	0.17	0.15	0.38	1.33	0.70	0.79
HORVU.GOLDEN_PROMISE.PROJ.6HG00535040.1	cultivar	view alignment	0.66	0.50	0.78	1.53	1.99	1.95	0.50	0.19	0.56	0.09	0.18	0.08	0.25	0.19	0.15
HORVU.HOCKETT.PROJ.2HG00164700.1	cultivar	view alignment	1.97	3.78	3.10	39.66	38.80	38.15	3.69	4.85	4.59	0.20	0.20	0.15	0.98	0.87	0.72
HORVU.HOCKETT.PROJ.6HG00532430.1	cultivar	view alignment	0.94	1.42	1.23	4.52	7.73	8.76	0.57	1.35	1.84	0.08	0.26	0.59	0.19	0.12	0.16
HORVU.HOR_10350.PROJ.2HG00159540.1	landrace	view alignment	8.18	6.68	6.50	75.05	85.81	60.53	11.89	9.36	12.85	0.91	0.56	0.68	1.44	1.46	n/a
HORVU.HOR_10350.PROJ.6HG00520710.1	landrace	view alignment	0.53	0.70	0.50	13.30	8.58	10.20	1.93	1.47	2.43	0.07	0.18	0.83	0.14	0.12	n/a
HORVU.HOR_13821.PROJ.2HG00165960.1	landrace	view alignment	7.76	7.63	5.02	134.16	154.79	132.27	16.96	14.20	12.82	9.10	10.50	7.90	1.76	1.95	2.61
HORVU.HOR_13821.PROJ.6HG00532900.1	landrace	view alignment	2.02	1.20	1.08	9.96	10.64	9.86	2.59	2.25	2.32	1.33	1.04	2.25	0.22	0.41	0.53
HORVU.HOR_13942.PROJ.2HG00163340.1	landrace	view alignment	4.39	4.89	4.03	51.58	47.38	49.13	6.51	6.11	7.59	1.35	1.12	1.00	1.35	1.31	1.26
HORVU.HOR_13942.PROJ.6HG00529290.1	landrace	view alignment	0.86	0.66	0.47	0.62	0.88	1.34	0.43	0.30	0.39	0.46	0.88	2.24	0.15	0.10	0.26
HORVU.HOR_21599.PROJ.2HG00164590.1	landrace	view alignment	7.45	6.68	10.39	58.74	53.90	55.05	20.35	17.30	18.32	0.75	0.71	2.54	2.19	1.20	1.92
HORVU.HOR_21599.PROJ.6HG00533940.1	landrace	view alignment	2.00	2.38	2.58	5.63	6.80	5.99	2.76	3.61	5.19	1.60	1.30	0.82	0.29	0.30	0.31
HORVU.HOR_3081.PROJ.2HG00164000.1	cultivar	view alignment	4.58	4.30	3.99	76.18	64.18	58.13	16.02	9.17	10.06	0.49	0.36	0.55	1.03	1.69	2.41
HORVU.HOR_3081.PROJ.6HG00528000.1	cultivar	view alignment	1.13	0.75	0.51	8.97	9.28	11.39	5.86	2.57	4.60	0.49	0.53	0.24	0.21	0.28	0.31
HORVU.HOR_3365.PROJ.2HG00163280.1	landrace	view alignment	5.15	4.50	4.70	32.99	28.29	28.38	6.10	5.39	5.92	0.42	0.29	0.39	0.62	1.24	2.45
HORVU.HOR_3365.PROJ.6HG00526210.1	landrace	view alignment	0.82	1.06	0.79	1.54	1.97	1.78	0.50	0.60	0.97	0.13	0.15	0.09	0.24	0.27	0.35
HORVU.HOR_7552.PROJ.2HG00166460.1	landrace	view alignment	5.09	5.66	4.67	77.85	n/a	63.42	13.84	14.35	12.00	2.71	7.78	4.10	1.33	1.84	1.75
HORVU.HOR_7552.PROJ.6HG00540680.1	landrace	view alignment	1.12	1.29	0.67	11.30	n/a	11.68	2.94	5.96	2.70	0.40	0.96	1.93	0.10	0.14	0.25
HORVU.HOR_8148.PROJ.2HG00164380.1	landrace	view alignment	4.44	5.55	4.83	30.34	30.46	31.81	4.78	4.30	5.46	2.12	1.50	n/a	0.57	1.13	1.88
HORVU.HOR_8148.PROJ.6HG00535270.1	landrace	view alignment	1.69	1.43	1.51	8.37	10.31	13.97	2.96	1.87	3.35	1.93	1.06	n/a	0.14	0.06	0.25
HORVU.HOR_9043.PROJ.2HG00166000.1	landrace	view alignment	6.87	5.55	6.34	77.69	79.21	80.52	11.63	9.46	13.32	1.65	3.46	3.71	1.41	1.67	5.07
HORVU.HOR_9043.PROJ.6HG00544260.1	landrace	view alignment	1.43	1.54	1.33	10.00	10.31	9.76	1.75	1.63	2.29	2.50	2.36	1.99	0.19	0.40	1.17
HORVU.IGRI.PROJ.2HG00163660.1	cultivar	view alignment	6.22	6.11	4.96	23.69	26.39	24.07	6.02	6.65	8.65	0.71	2.01	0.29	0.93	0.68	1.12
HORVU.IGRI.PROJ.6HG00532270.1	cultivar	view alignment	0.98	0.92	1.04	5.87	10.54	7.43	0.46	1.87	1.88	0.20	1.45	0.29	0.17	0.22	0.38
HORVU.MOREX.PROJ.2HG00162860.1	cultivar	view alignment	8.32	13.06	14.13	42.73	37.17	29.03	5.29	5.23	6.70	0.59	0.71	0.52	1.65	1.59	2.51
HORVU.MOREX.PROJ.6HG00528800.1	cultivar	view alignment	1.48	0.65	0.76	0.26	0.72	0.50	0.10	0.07	0.08	0.96	0.24	2.16	0.25	0.48	0.24
HORVU.OUN333.PROJ.2HG00166980.1	landrace	view alignment	5.58	7.90	7.18	26.21	27.53	28.60	9.52	7.87	8.91	0.83	1.08	2.98	2.61	1.75	1.59
HORVU.OUN333.PROJ.6HG00536560.1	landrace	view alignment	1.43	0.95	1.12	10.47	15.24	14.60	3.95	3.39	2.92	0.94	1.28	1.08	0.33	0.08	0.32
HORVU.RGT_PLANET.PROJ.2HG00163840.1	cultivar	view alignment	2.76	3.99	3.16	18.16	19.51	20.91	3.41	3.70	4.13	0.30	0.98	1.05	1.11	1.54	1.20
HORVU.RGT_PLANET.PROJ.6HG00534460.1	cultivar	view alignment	2.01	2.22	2.06	5.24	6.82	6.73	0.36	0.66	0.61	0.22	0.72	0.87	1.22	0.52	0.30
HORVU.ZDM01467.PROJ.2HG00163660.1	landrace	view alignment	11.12	8.29	8.42	47.47	49.21	33.20	8.31	8.11	6.99	6.99	16.54	1.34	5.16	5.16	5.32
HORVU.ZDM01467.PROJ.6HG00532930.1	landrace	view alignment	1.19	1.94	1.49	0.91	0.74	0.42	0.15	0.25	0.22	1.68	5.65	0.72	0.50	0.25	0.26
HORVU.ZDM02064.PROJ.2HG00165940.1	landrace	view alignment	6.23	8.32	8.03	38.99	34.62	36.10	7.65	6.97	8.45	1.91	6.04	2.60	1.45	1.06	1.34
HORVU.ZDM02064.PROJ.6HG00535880.1	landrace	view alignment	1.82	2.40	1.82	1.16	1.34	1.14	0.65	0.40	0.39	1.73	2.16	1.72	0.12	0.10	0.19
Mean TPM per tissue across all genes and replicates				*3.82*			*27.52*			*5.11*			*1.65*			*0.97*	

Figure S2. Screenshot of the gene expression profile of Candidate gene HORVU2Hr1G092290/ HORVU.MOREX.r3.2HG0184740 (A) and HORVU2Hr1G092460/HORVU.MOREX.r3.2HG0184930 (B), for 20 barley pangenome accessions taken from the PanBARLEX database (https://panbarlex.ipk-gatersleben.de/). Expression was measured for five tissue types (Embryo, Root, Shoot, Inflorescence and Caryopsis). The expression level is provided as 'Transcripts per Million' (TPM), with the mean TPM provided across all genes/replicates at the bottom of each tissue category.

Figure S3. Distribution of absolute difference values for the trait of 'Average grain number per spike' calculated for duplicated plots (n = 36). The outlier threshold of 5 grains is labelled below.

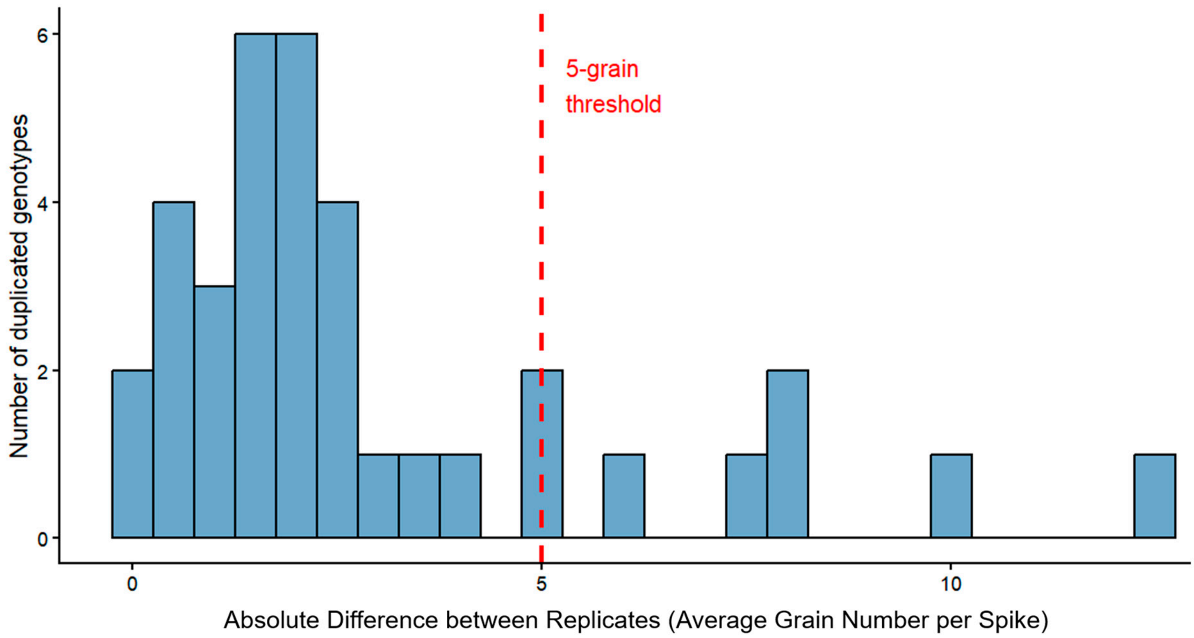

Supplement: Supplementary file 1 [file plants-15-01518-s001.zip › plants-4282694-supplementary.pdf]
